# The Gap of Health Inequalities Amongst Lung Cancer Patients of Different Socioeconomic Status: A Brief Reference to the Greek Reality

**DOI:** 10.3390/cancers16050906

**Published:** 2024-02-23

**Authors:** Amalia Sofianidi, Alexandra Karadimou, Andriani Charpidou, Konstantinos N. Syrigos

**Affiliations:** Oncology Unit, Third Department of Internal Medicine, Sotiria General Hospital for Chest Diseases, National and Kapodistrian University of Athens, 11527 Athens, Greece; alexandra.karadimou@yahoo.gr (A.K.); dcharpidou@yahoo.gr (A.C.); ksyrigos@med.uoa.gr (K.N.S.)

**Keywords:** lung cancer, socioeconomic status, education, incidence, prognosis, stigma

## Abstract

**Simple Summary:**

Lung cancer treatment and patient care continue to advance, yet concerns persist about whether these improvements are equally accessible to all socioeconomic groups. Socioeconomic disparities exist in lung cancer incidence, screening, effective treatment, overall survival, and prognosis. One of the key contributing factors to low socioeconomic status that is amenable to change is low education. Lower educational attainment is oftentimes linked to various factors, including smoking habits, unhealthy lifestyle behaviors, lower paid and unhealthier occupations, exposure to environmental pollutants, and genetic-familial risks, all contributing to an elevated incidence of lung cancer. Driven by the observed health inequalities within the Greek population, the Greek Society of Lung Cancer has launched the “MIND THE GAP” campaign, which aims to raise awareness and narrow the gap associated with lung cancer, not only in Greece but across Europe. The aim of this literature review is to explore the gap of health inequalities regarding lung cancer incidence and prognosis between patients of different SES and its root of causality. Key pivotal actions towards bridging this gap are reviewed as well.

**Abstract:**

Lung cancer treatment and patient care are constantly improving, but it remains doubtful whether this applies equally to all socioeconomic groups. It is nowadays well established that there are socioeconomic inequalities regarding lung cancer incidence, screening, effective treatment, overall survival, and prognosis. One of the key contributing factors to low socioeconomic status is low education. Low educational level is correlated with several factors, such as smoking habits, bad lifestyle behaviors, lower paid and unhealthier occupations, polluted neighborhoods, and genetic-familial risk, that lead to increased lung cancer incidence. The disparities regarding lung cancer care are further enhanced by stigma. On this basis and inspired by the gap in health equality among the Greek population, the Greek Society of Lung Cancer initiated a campaign, “MIND THE GAP”, to help increase awareness and minimize the gap associated with lung cancer, both in Greece and across Europe. The aim of this review is to explore the gap of health inequalities regarding lung cancer incidence and prognosis between patients of different SES and its root of causality. Key pivotal actions towards bridging this gap are reviewed as well.

## 1. Introduction

Lung cancer ranks as the second-most frequently diagnosed malignancy in both men and women. However, it stands as the foremost cause of cancer-related mortality worldwide and surpassed the combined fatalities from breast, colorectal, and prostate cancers in 2020 [1]. This burden disproportionately affects people with lower socioeconomic status (SES). One of the first articles about the disparities regarding lung cancer was published in 1958, where it was stated that there was an increase in lung cancer mortality related to inequalities in different social classes and occupations [2]. Nowadays, it is possible that the existing inequalities are even greater as a result of the COVID-19 pandemic, which suddenly changed our routines, modified our health behaviors, and significantly disrupted the landscape of lung cancer care. More specifically, the stress associated with COVID-19 and the economic crisis was linked to an increase in smoking, predominantly in individuals from populations especially vulnerable to smoking. In addition, the pandemic lockdown resulted in delaying screening, diagnosis, and treatment of many cancers and in putting a halt to clinical trials [3].

According to the WHO, the social determinants of health are the nonmedical factors that influence health outcomes. These are the circumstances in which individuals come into existence, develop, work, reside, and age. They encompass the broader array of influences and systems that mold the conditions of everyday life. Studies indicate that social determinants play a more significant role in influencing health than healthcare alone. For instance, multiple research findings propose that social determinants of health contribute to approximately 30–55% of health outcomes. Social determinants of health play a crucial role in the existence of health inequities, which refer to unjust and preventable differences in health status observed within and among nations. Regardless of income level, a social gradient is evident in health outcomes, where individuals in lower social standing positions experience poorer health.

There are many factors that are related to low SES, such as education, income, environment, and occupation. All factors are connected to one another and influence each other. The key contributing and amenable-to-change factor is low education. Individuals with lower education levels often have limited access to resources such as information, healthcare services, and preventive measures, ignoring the importance of health literacy. Lower education levels may lead to employment in occupations with higher physical risks, exposure to environmental hazards, and lower job security, all of which can impact health negatively. Also, limited educational attainment is associated with low-pay occupations or unemployment that may contribute to chronic stress, which can adversely affect mental health and, in turn, impact physical health and exacerbate existing health conditions. Moreover, low income also relates to inhabiting environmentally degraded and unhealthy neighborhoods.

A systematic search of PubMed, alongside Google Scholar, was performed using keywords related to low SES, lung cancer, health inequalities, and Greece, with emphasis on papers published in the last decade. The aim of this literature review article is to present SES as a risk factor for lung cancer and to assay and determine the key contributor and amenable-to-change factor, in order to promote efforts towards closing the gap of health inequalities. A concise reference to the Greek reality will be considered as well.

## 2. Lung Cancer Incidence and SES

Several studies have shown an association between SES and incidence of lung cancer. Interestingly, this association is observed not only in underdeveloped countries but also in countries with excellent health systems. A registry-based study in Norway investigated the correlation between socioeconomic factors—utilizing income and education as indicators—and cancer incidence in Norway, a country recognized for its egalitarian principles, universal healthcare access, and high human development index. The most significant variations in incidence rate ratios (IRR) were observed in lung cancer. Individuals, both men and women, with a college or university education as their highest completed level showed a two- to threefold reduction in risk compared with those with primary school education (IRR for men: 0.40 (0.37–0.43), for women: 0.34 (0.31–0.37)) [4]. Low educational level is correlated with several factors, such as smoking habits, bad lifestyle behaviors, polluted neighborhoods, and genetic-familial risk, that lead to increased lung cancer incidence.

### 2.1. Smoking Habits

The link between active tobacco use and exposure to secondhand smoke in causing lung cancer is firmly established, with tobacco exposure contributing to approximately 80–95% of lung cancers [5]. Smoking continues to be the primary cause of avoidable illness and death on a worldwide scale [6], despite significant public health initiatives that have had a substantial impact on decreasing the prevalence of cigarette smoking. Nevertheless, smoking is not evenly spread throughout society. Instead, it is progressively clustering among individuals with the lowest education, income, and occupational status. As a result, it plays a pivotal role in contributing to health disparities, representing a significant portion of the variations in lung cancer incidence and mortality linked to SES [7,8].

Education stands out as one of the most robust sociodemographic indicators for predicting smoking prevalence and cessation. Lower education is associated with elevated smoking prevalence and reduced cessation rates [9]. An article published in JAMA in 1989 showed that, from 1974 to 1985, education took precedence over gender as the most influential sociodemographic factor associated with smoking [10]. The trend is the same 35 years later, with the prevalence of smoking being much higher among individuals without a high school diploma compared with those with a college degree [9]. In fact, marketing plays a big role in whether people try or use commercial tobacco products. The tobacco industry directs its marketing and advertising efforts towards communities with lower incomes and lower health literacy status. In the United States, it has been demonstrated that two major tobacco manufacturers specifically tailor their advertising to attract “working-class” youth. This further contributes to the initiation of smoking among young individuals from lower socioeconomic backgrounds at an earlier age [11] and leads to lower screening rates, increased diagnosis at advanced-stage disease, and lower rates of surgical intervention for early-stage disease observed in low SES communities [12].

In previous years, lower education was also correlated with lower rates of quitting smoking [13]. Minorities are less prone to receiving guidance to quit smoking, possessing awareness of accessible smoking cessation programs, engaging in smoking cessation initiatives, or utilizing pharmacotherapy to cease smoking [14]. However, recent data from the CDC state that people with lower incomes and less education try to quit using tobacco similarly to other sociodemographic groups. In 2015, an estimated 50% of adults who smoke cigarettes, and who also have not earned a high school diploma, tried to quit smoking, compared with 58% of those with some college education [15]. Notably, smokers in the lower socioeconomic class require additional support and assistance in quitting smoking, given that they are frequently more deeply addicted and more frequently exposed to nicotine [16].

The most recent globally supported data correlating low educational status with smoking and increased lung cancer incidence are from an analysis of data from 15 countries in the Lung Cancer Cohort Consortium. In both current and former smokers, a higher level of education demonstrated a remarkably consistent reduction in the risk of developing incident lung cancer across cohorts spanning four continents. This pattern persisted even after thorough adjustments for smoking were taken into account. When grouping by world region, the association between education and lung cancer incidence among currently/formerly smoking participants was similar for the US (hazard ratio (HR) = 0.88, 95% confidence interval (CI) 0.87–0.89), Europe (HR 0.89, 95% CI 0.88–0.91), and Asia (HR 0.91, 95% CI 0.86–0.96), but attenuated in the Australian cohort (HR 1.02, 95% CI 0.95–1.09 in all cohorts), except in the US Southern Community Cohort Study. This particular study, predominantly consisting of African Americans, exhibited a hazard ratio of 0.75 (95% CI 0.62–0.90) [17].

### 2.2. Environmental and Occupational Exposure

According to a study by the World Health Organization (WHO), maintaining healthy working and living environments has the potential to prevent at least 1.7 million cancer deaths each year [18]. While smoking is the main cause of lung cancer, there are other environmental risk factors that contribute to the pathogenesis of lung cancer [19]. These factors contribute to the pathogenesis of lung cancer in nonsmokers or intensify the deleterious effects of smoke on the lung [20]. The International Agency for Research on Cancer (IARC) has categorized air pollution as a Group 1 carcinogen, meaning it is confirmed to be carcinogenic to humans. This classification, based on IARC’s 2013 findings, is associated with over 220,000 annual global deaths from lung cancer and is linked to reduced survival rates post-diagnosis [21,22]. At the ESMO Congress in 2022, it was reported that very small pollutant particles in the air may trigger lung cancer in people who have never smoked. These particles, which are typically found in vehicle exhaust and smoke from fossil fuels, are associated with non-small cell lung cancer (NSCLC) risk and drive mutations in EGFR and KRAS genes [23].

Education and the environment are strongly connected to one another. It is well established that populations with lower SES are more likely to be exposed to higher levels of air pollution than those with higher SES [24,25,26]. Individuals with lower incomes may find themselves residing in more affordable regions where land values have decreased, such as areas near major roadways or industrial zones. This can result in heightened exposure levels and, consequently, an increased risk of disease. The issue of indoor air pollution stemming from combustion byproducts is especially worrisome in developing nations and rural areas, where wood and charcoal are frequently utilized for cooking and heating. Research indicates that implementing proper ventilation in these cooking spaces can lead to a reduction in lung cancer risk of up to 50% [27].

Education can influence individual behaviors and attitudes towards the environment. Environmental education refers to the adoption of educational methods aimed at fostering citizens’ understanding of their ethical connection to the environment, enhancing their awareness of environmental protection, skills, attitudes, and values, and guiding them to prioritize environmental concerns and undertake actions conducive to fostering a process of civic education conducive to sustainable development. Individuals with lower levels of education may have limited awareness and understanding of environmental issues, including climate change, pollution, and resource depletion. This lack of awareness can contribute to unsustainable behaviors and practices that harm the environment. On the same basis, individuals with low education levels may lack the necessary skills to work in environmentally friendly industries or to adopt sustainable practices in their daily lives, such as energy efficiency or waste reduction [28].

Asbestos is also carcinogenic and can cause lung cancer and mesothelioma [29]. People from lower SES are more likely to work in mines, being exposed to asbestos every day. Numerous epidemiological studies have been conducted on workers exposed to asbestos [30], but there is a limited number of studies focusing on the health effects of asbestos exposure in household and residential settings. Primary concerns regarding household exposure involve the immediate family members of asbestos workers, stemming from dust brought home on clothing. Household sources of asbestos exposure include the degradation, installation, removal, and repair of products containing asbestos. Radon exposure is also associated with an increased risk of lung cancer. The duration spent in subterranean environments, such as basements or mines, particularly in regions with elevated uranium concentrations, is linked to radon exposure. Individuals working underground in metal or uranium mining are recognized to face a significantly heightened risk of squamous cell carcinoma in the lungs and other organs [31,32,33].

In France in 2015, lung cancer stood out as the predominant form of cancer linked to occupational exposures. Among men, there were 5621 cases (constituting 89% of all work-related cases), while among women, there were 294 cases (making up 80%) [34]. Many epidemiological studies conducted in various countries have identified a notably elevated mortality rate for lung cancer among construction workers [35,36,37,38]. Construction workers face an increased risk of developing lung cancer with prolonged exposure, a risk not evident in supervisors, engineers, and higher-ranking officials in construction. This distinction is attributed to the lower exposure levels of the latter group compared with those working directly on construction sites. An analysis of data from a multicenter case-control study of lung cancer conducted in six Central and Eastern European countries showed that exposures in the workplace, such as to diesel exhaust and welding fumes, and to a lesser extent, crystalline silica, contribute to the risk associated with educational levels in the development of lung cancer. Between 13.4% and 14.8% of the association between education and lung cancer is mediated by occupational exposure [39]. Another French analysis showed that considering socio-occupational groups, the aggregate attributable fraction for three occupational carcinogens (asbestos, silica, diesel motor exhaust) reached 26.7% (95% CI 22.5; 30.8) for blue-collar workers, whereas it was minimal, at 0.2% (95% CI −1.35; 1.64), for managers [34]. In other words, it was proven that a portion of the increased likelihood of lung cancer among individuals with lower educational levels is attributed to their occupational exposure to carcinogens.

### 2.3. Genetic Predisposition

Genome-wide association studies (GWAS) have identified variants in multiple chromosomal regions linked to an elevated hereditary risk of lung cancer. These encompass the 5p15 locus, housing the gene for telomerase reverse transcriptase (TERT) [40], the 6p21 locus, responsible for regulating G-protein signaling [41], and the 15q25–26 loci, demonstrated to enhance nicotine dependence and susceptibility to lung cancer [42]. A study conducted in China explored the potential causal association between an increased number of years in education and a reduced risk of lung cancer, utilizing a two-sample Mendelian randomization (MR) study. It was demonstrated that genetically predicted higher educational attainment was related to significantly lower odds of lung cancer. Using conventional MR analysis, one standard deviation (SD) of longer education, specifically 3.6 years of additional education (due to genetic predisposition across 73 single nucleotide polymorphisms), was associated with a 52% lower risk of lung cancer [odds ratio (OR) 0.48, 95% CI 0.34, 0.66, *p* = 1.02 × 10^−5^]. A genetic inclination towards extended education was also linked to reduced smoking, a lower body mass index, and a favorable blood lipid profile [43].

### 2.4. Familial Socioeconomic Position

The socioeconomic status of the family during childhood plays a crucial role in predicting certain chronic diseases. Researchers in Denmark tried to investigate whether familial factors shared among siblings account for the correlation between education and the risk of lung cancer. Using the valid information of millions of siblings born in Denmark between 1950 and 1979, it was shown that family factors shared by siblings, such as exposure to secondhand smoke from parents, confounded some of the association between education and lung cancer incidence [44]. Wang et al. performed a meta-analysis of 13 studies in order to investigate whether childhood SES, defined as the education level or socioeconomic position of parents, and/or childhood housing conditions, influenced lung cancer mortality. Poorer childhood SES was associated with increased lung cancer risk (HR 1.25, 95% CI 1.10, 1.43) [45]. However, this meta-analysis was still in the preprint stage and had not been externally reviewed.

### 2.5. Infections

The process of lung carcinogenesis has been associated with inflammation and cellular damage occurring during respiratory infections. Tuberculosis (TB) increases the risk of lung cancer onset and progression according to a meta-analysis, expressing an OR of lung cancer development of 1.76 [46]. On the other hand, HIV also increases the risk of lung cancer by two to four times, regardless of smoking status [47]. In fact, in the US, lung cancer has emerged as the primary cause of mortality among individuals with HIV, especially after the introduction of antiretroviral therapies that limited the incidence of AIDS-defining infection [47,48,49]. Patients infected with TB and HIV are more likely to be minorities, of low SES, and smokers.

**Figure 1:**
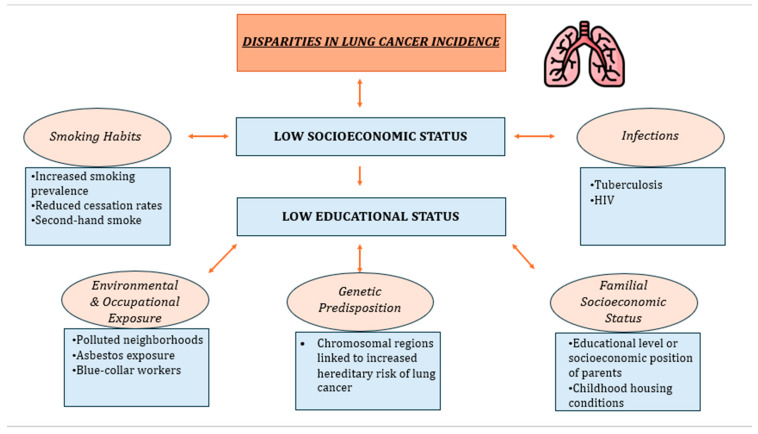
The key contributing factor to low SES is low education. Low educational level is associated with several factors, such as smoking habits, bad lifestyle behaviors, polluted neighborhoods, and genetic-familial risk, that lead to increased lung cancer incidence.

Figure 1 provides a schematic presentation of the factors that contribute to lung cancer health disparities.

## 3. Relation between Low SES and Poor Lung Cancer Prognosis

### 3.1. Screening

Lung cancer screening with the use of low-dose computed tomography (LDCT) is nowadays well established as a tool to detect the disease early, improve the chances of successful treatment, and reduce mortality rates [50,51]. The National Lung Screening Trial (NLST), a randomized trial that compared the outcomes of smokers who were screened via LDCT versus (vs.) those who were screened via chest X-ray for several consecutive years in a row, showed a 20% reduction in non-small cell lung cancer (NSCLC) mortality and a 6% reduction in all-cause mortality when using LDCT as a screening method [50,52]. The updated 2023 guidelines by the American Cancer Society recommend annual lung cancer screening with LDCT for asymptomatic individuals aged 50–80 years who currently smoke or formerly smoked and have a ≥20 pack-year smoking history [53]. Unfortunately, the research conducted over the years informing the guidelines for lung cancer screening did not prioritize the inclusion of individuals from diverse socioeconomic backgrounds, overlooking the comparatively lower survival rates among those with limited financial resources. A narrative review of lung cancer screening socioeconomic disparities by Castro et al. demonstrated that the majority of participants in lung cancer screening studies were of higher SES, and the percentage of participants with a college degree or higher (32%) was more than double the percentage among individuals in the general population who met NLST age and smoking history inclusion criteria (14%) [54]. It is more than obvious that higher SES groups are overrepresented in lung cancer screening studies [55].

The lower utilization of lung cancer screening among individuals with lower individual and median household incomes is likely attributed to various factors, including financial barriers. Most patients with low SES have no health insurance. An individual with low income and no insurance would have to bear the cost of a scan from their own funds, a scenario that is unlikely to occur, given various competing financial priorities. Therefore, the type of insurance and its coverage can influence the rates of utilization among individuals with low SES [54]. Moreover, individuals with lower incomes are more prone to holding jobs that lack flexibility in accommodating lung cancer screening during the hours when radiology centers are ordinarily open [54].

Patients with low SES lack the necessary health literacy to understand the benefits and the purpose of lung cancer screening. Williams et al. tried to measure knowledge, awareness, decisional conflict, preferences, and values related to lung cancer screening. The study identified slight associations between higher levels of education and increased knowledge scores regarding lung cancer screening (*p* = 0.06), as well as an association between lower education levels and the perception of more disadvantages in undergoing lung cancer screening (*p* = 0.09) [56]. Multiple studies also show that a patient’s enhanced comprehension of their treatment plan and, consequently, higher rates of lung cancer screening, can be supported by attaining a high level of education and health literacy [57,58].

Transportation barriers to screening centers also contribute to the lower lung cancer screening rates observed in low-income communities. Transportation barriers correlated with geographic barriers as well. A study in Missouri and Illinois investigated how living in rural areas affects lung cancer screening. Compared with 41% of nonmetropolitan residents, approximately 98% of metropolitan residents had access to screening. However, lung cancer mortality in rural residents is multifactorial and cannot be explained by access alone [59].

Sosa et al. examined disparities in lung cancer screening related to socioeconomic factors, specifically concentrating on six studies conducted exclusively in the United States [60]. This review showed some evidence for socioeconomic disparities in lung cancer screening in the US, although the number of included studies was inadequate. Reduced household income was linked to a decreased likelihood of meeting the criteria for screening eligibility in one study. There was no clear effect of educational level on screening eligibility in two studies. In two out of three studies, it was demonstrated that lower-income individuals were less likely to complete screening or have an intention to be screened. There was no difference in stage at the time of diagnosis between different SES individuals, as assessed in another study. Overall, this review shows some level of socioeconomic disparities in lung cancer screening in the US, but more studies are needed to further explore this observation.

As far as Europe is concerned, lung cancer screening using LDCT has not been widely adopted or implemented [61]. As a result, no actual conclusions can be drawn regarding lung cancer screening disparities in Europe. On this basis, the EU Cancer Inequalities Registry was created to pinpoint trends, disparities, and inequalities across member states and regions. In addition to ongoing qualitative evaluations of the situation specific to each country, the registry will identify challenges and specific areas requiring action. This will help direct investment and interventions at the EU, national, and regional levels as part of Europe’s Beating Cancer Plan [62].

### 3.2. Effective Treatment

Lung cancer treatment options range from traditional therapies, such as surgery, chemotherapy, and radiotherapy, to more advanced treatment modalities, such as immunotherapy and more targeted treatment options. Forrest et al. tried to investigate whether SES was related to the receipt of traditional treatment in lung cancer patients [63]. As expected, lower SES was associated with a lower likelihood of receiving any type of treatment (OR 0.79 (95% CI 0.73–0.86), *p* < 0.001). Regarding various treatment approaches, individuals with lower SES exhibited a reduced likelihood of undergoing surgery (OR 0.72 (95% CI 0.65–0.80), *p* < 0.001, even when adjusting for histology and stage at diagnosis). Additionally, for chemotherapy, the OR was 0.81 (95% CI 0.73–0.89, *p* < 0.001), and for unspecified treatment (cases where the specific type of treatment was not reported), the OR was 0.78 (95% CI 0.74–0.83, *p* < 0.001). There was no discernible association between SES and the likelihood of receiving radiotherapy (OR 0.99, (95% CI 0.86 to 1.14), *p* = 0.89). However, a narrative review by Lin et al. showed that there are indeed socioeconomic disparities in the use of stereotactic body radiation therapy (SBRT) for treating non-small cell lung cancer (NSCLC). SBRT is increasingly recognized as a viable and effective alternative for NSCLC patients unable to undergo surgery due to pulmonary or cardiovascular limitations, other comorbidities, or personal preference [64,65,66]. Overall, patients with low income and education levels, and from nonmetropolitan areas, were less likely to receive SBRT, which correlates with worse clinical outcomes [67]. Another large cohort in the US including 69,168 patients with stage I NSCLC conducted by the National Cancer Database supports that, for patients with multiple SES risk factors, the odds of receiving nonstandard treatments, such as chemotherapy and radiation therapy, linearly increase, while the odds of receiving no therapy quadratically increase. For patients with five factors (low income, non-White race, low high school graduation rate, Medicaid or no insurance, rural residence, and distance less than 12.5 miles from treatment facility), the OR is 4.7 (95% CI 3.44–6.30) [68].

However, the future of lung cancer treatment lies in precision oncology, which aims to revolutionize cancer care by identifying and treating the characteristics of an individual’s cancer [69]. Advancements in precision oncology are primarily driven by technological advances in genomic sequencing, such as next-generation sequencing (NGS) [70], which leads to targeted therapies for specific mutations in lung cancer [71]. The question here is whether precision oncology has the potential to further widen disparities regarding lung cancer treatment and overall survival. This issue is highlighted by the fact that certain vulnerable populations are underrepresented in clinical trials and have limited access to NGS [72]. A meta-analysis by Norris et al. tried to investigate whether there are socioeconomic inequalities in the utilization of predictive biomarker tests and/or biological and precision lung cancer therapies [73]. It is worth noting that all of the identified studies were conducted in the United States. There was no correlation between SES and the use of predictive biomarker tests (specifically, EGFR and/or ALK): OR 0.92 (95% CI 0.35–2.40). Nevertheless, lower SES was associated with reduced utilization of biological and precision therapies: OR 0.71 (95% CI 0.51–1.00). An analysis of the National Cancer Database in the US regarding socioeconomic disparities in immunotherapy use among advanced-stage NSCLC patients confirmed our previous observation. Genetic testing is often required before immunotherapy can be provided [74], and socioeconomic inequalities regarding genetic testing have been observed as well [75]. On this basis, it was demonstrated that disparities in the administration of immunotherapy based on income and education levels are evident among non-Hispanic White patients receiving treatment in both academic and non-academic healthcare settings. Similar patterns were noted among non-Hispanic Black patients concerning area-level education, though statistical significance was only reached for those treated at non-academic facilities. Academic healthcare facilities generally have greater resources than community facilities, providing patients with increased access to specialists and potentially leading to better outcomes, irrespective of socioeconomic resources [76].

Because of its aggressive nature, individuals with small cell lung cancer (SCLC) may encounter a range of symptoms that can be alleviated with the support of palliative care. Palliative care can improve a person’s quality of life. Disparities regarding the utilization of PC have also been observed. The receipt of palliative care by individuals with SCLC is influenced by factors such as facility type, location, insurance, income, gender, age, and residential area. Among patients in the US who receive palliative care, 78% live in metropolitan areas, while only 2.8% live in rural areas. Patients undergoing treatment at facilities in the Mountain (2.8%), Pacific (5.7%), and West South Central (5.6%) regions are less likely to receive palliative care (*p* < 0.05) [77].

Recently, Patel et al. tried to investigate whether lung cancer disparities might be in part due to differences in microbiome diversity determined by the unhealthy dietary patterns dictated by lower SES. It is nowadays well established that the human microbiome can predict responsiveness or resistance to immunotherapies involving immune checkpoint inhibitors (ICIs) and other therapeutic modalities [78,79]. Higher SES index patients had elevated fecal levels of Actinobacteria, Firmicutes, and Proteobacteria, and lower levels of Bacteroides compared with the Human Microbiome Project’s (HMP) reported normal levels. Further exploration is needed to investigate the connections between SES and microbial distribution in these patients [80].

### 3.3. Overall Survival and Prognosis

According to the American Cancer Society, the overall 5-year relative survival rate is 28% for NSCLC and 7% for SCLC [81]. Unfortunately, patients from lower SES have even lower survival rates, not only because of the disparities regarding their treatment options but also due to the increasing number of advanced-stage and more aggressive diagnoses. Individuals with lower SES seem to have a higher likelihood of developing and succumbing to lung cancer compared with those with a high SES [82]. An analysis of the National Cancer Database shows that lower area-level education and income are associated with higher odds of an advanced-stage NSCLC diagnosis, irrespective of the type of facility and for individuals with both government and private insurance coverage (OR for education 1.12, 95% CI 1.10–1.13, OR for income 1.13, 95% CI 1.11–1.14) [83]. In Maryland, lower block-group social class and increased expenditure on tobacco were linked to aggressive lung cancer types, such as squamous and small cell histological types, as well as poorly differentiated or undifferentiated tumor grades [84].

Overall, individuals with private insurance, Medicaid, Medicare, or other government insurance are all less prone to being diagnosed with advanced-stage cancer than those without insurance. Individuals in the second and fourth quartiles tend to receive an earlier diagnosis in comparison with patients in the lowest median household income quartile. Moreover, patients residing in areas where a higher percentage of residents without a high school diploma are more likely to be diagnosed with advanced NSCLC [85]. The same results are observed in SCLC patients [86,87].

The Canadian Partnership Against Cancer works with and supports Canada’s health and cancer community to help improve cancer outcomes. A recent report states that people with lower income and people who live in rural and remote communities are 13–25% less likely to survive 3 years, depending on stage at diagnosis [88].

### 3.4. Stigma

The disparities regarding lung cancer care are further enhanced by stigma. The stigma associated with lung cancer is a multifaceted phenomenon influenced by various factors at different levels, encompassing the perspectives of the patient, the clinician, and society. Unfortunately, the notable increase in awareness and treatment for lung cancer has not been matched by a corresponding decrease in the stigma associated with the disease [86], with patients from lower SES being disproportionately affected. As far as society is concerned, 60% thinks that smokers with lung cancer are at least partially to blame for their disease, 27% thinks that nonsmokers who get lung cancer should get their treatment prioritized over those who smoke, and 17% thinks that health systems should not cover lung cancer patients who smoke. As far as the patients are concerned, 30% blame themselves for their disease, 42% feel less deserving of help, 45% put off doctor visits because of self-blaming, and 55% socially isolate because of blame and shame [87]. It is obvious that lung cancer stigma continues to pose a challenge across the cancer care community, further promoting inequalities in the lung cancer care continuum.

**Figure 2:**
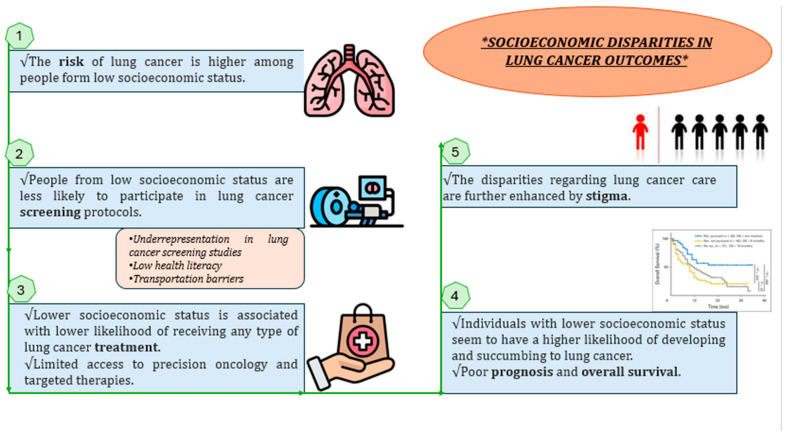
A general summary of the evidence available regarding inequalities in lung cancer outcomes, including incidence, screening, treatment, overall survival, and prognosis. The disparities regarding lung cancer care are further enhanced by stigma [54,63,73,82].

A summary of how socioeconomic disparities affect lung cancer outcomes is presented in Figure 2.

## 4. Lung Cancer in the Greek Reality

Lung cancer is the most commonly diagnosed malignancy in Greece, accounting for over 13.9% of total new cases and being the leading cause of cancer-related mortality in 2020 (23.1% of deaths) [89]. Equal health provision remains a challenge in Greece, not only because of the financial deficit crisis that escalated in 2009 but also because of the unique anthropogeography of Greece, with many large and numerous small islands that are not easily accessible, especially during the winter months. Access to healthcare, encompassing factors such as proximity to medical facilities, ratio of physicians to the population, and availability of cancer detection technologies and screening methods, represent key components of social deprivation and rural and island living. According to the EU Cancer Inequalities Registry, socioeconomic determinants of health are important drivers of the cancer burden in Greece.

There is a scarcity of research examining the attributes of smoking and tobacco exposure specifically within rural populations in Greece. The elevated prevalence of smoking and the heightened levels of tobacco exposure indicate a population at elevated risk for diseases associated with tobacco use [90].

Despite its high epidemiological burden, Greece does not implement a national lung cancer screening strategy. There is no official recommendation from the Ministry of Health regarding low-dose CT, but there are efforts for lung cancer screening programs in high-risk populations in some large hospitals in Athens, Thessaloniki, and Crete on the basis of the scientific results of the National Lung Screening Trial [50] and NELSON trials [51]. Unfortunately, rural Greek areas, as well as the Greek islands, are basically excluded from these efforts due to transportation issues. A study published in 2022 by Souliotis et al. revealed that applying a 100% screening strategy among high-risk adults aged 50–80 would result in additional averted deaths and lung cancer life years gained over 5 years in Greece [91].

As far as the diagnosis of lung cancer is concerned, the National Insurance Authority financially supports the molecular analysis for EGFR, ALK, BRAF, and KRAS genetic alterations. The detection of PD-L1 is not funded by national insurance but is sometimes available through diagnostic programs sponsored by pharmaceutical companies. EBUS (endobronchial ultrasound) is a valuable technique for obtaining tissue samples and staging lung diseases, providing a minimally invasive way to access and evaluate lesions in the lungs and surrounding areas. The availability of proper tissue sampling and staging faces constraints in Greece due to the limited presence of EBUS systems in public hospitals and the scarcity of pulmonologists with specialized training in this technique [92].

As far as diagnostic imaging techniques are concerned, CT and magnetic resonance imaging services are readily accessible throughout Greece. However, facilities for bone scans are primarily concentrated in larger islands and urban centers in continental Greece. The availability of PET/CT scans is limited to nine major academic, public, and private hospitals located exclusively in Athens and Thessaloniki. This situation, coupled with the absence of EBUS, necessitates that numerous patients relocate from their residences to major urban centers to undergo comprehensive diagnostic and staging procedures [92].

Radiotherapy as a treatment modality in Greece is severely hindered. Presently, there are 27 radiotherapy centers situated in all major urban regions across continental Greece, but there are none in the islands (with the exception of Crete, which is the largest Greek island). The geographical distribution of radiotherapy facilities remains a significant concern in Greece. Residents of Greek islands are required to relocate to either continental Greece or Crete for several weeks to access the necessary radiotherapy care. As a result, besides transportation issues, there are long waiting times for radiotherapy initiation in the public healthcare system [92]. Even in 2010, Hillas et al. demonstrated that a disparity of health facilities in an urban area discourages proposed treatment application in inoperable lung cancer patients. In Greece, it is common to observe the absence of a radiotherapy department in a hospital that administers chemotherapy, which hinders the implementation of prevailing guidelines supporting combined radiochemotherapy. In instances where radiotherapy is advised following six cycles of chemotherapy, half of patients express reluctance to undergo displacement and consequently do not adhere to the recommendations. This reluctance has repercussions on patient survival [93].

In terms of clinical and translational research in Greece, enhancing funding for translational and clinical research in lung cancer, as well as fostering international collaborations, can facilitate improved patient access to relevant clinical trials [92].

In 2023, a study organized by the Hellenic Society of Medical Oncology tried to explore the use of a digital platform for cancer patients to self-report their demographics, disease and therapy characteristics, and socioeconomic issues. The aim of this innovation was to improve cancer care by improving communication between cancer patients and physicians through the elimination of transportation barriers and by reducing the risk of infection, hospital visits, paperwork, and clinical burden. The most commonly reported cancers were lung and breast cancer. Age, education, and socioeconomic disparities were, however, shown to limit the use of digital health innovation. Older people and people of lower education were less likely to participate in this program [94].

## 5. Discussion

SES is an indicator of an individual’s standing within societal structures, typically evaluated through the interconnected factors of education, occupation, and income. Several studies have established the correlation of low SES with increased lung cancer incidence and poor lung cancer prognosis.

In an effort to prevent lung cancer from becoming the next global public health emergency, the Consensus Statement for Bridging the Gap in the Diagnosis and Management of Lung Cancer was presented to and ratified by the attendees of the 9th International Lung Cancer Network in June 2023. This consensus statement reaffirms the dedication to addressing five overarching principles that can serve as a common ground for all stakeholders. These stakeholders include health and public health professionals, policymakers, and government departments in health, finance, social services, and education, as well as patients and patient organizations. Together, they can collaborate to formulate and enhance policies aimed at improving outcomes for individuals at risk of and living with lung cancer. The five fundamental principles encompass enhancing and broadening prevention efforts, emphasizing early detection, ensuring fair and sustainable access to treatment, fostering partnerships and encouraging investment, and addressing stigma as a recognized social determinant of public health.

On this basis and inspired by the gap in health equality among the Greek population, the Greek Society of Lung Cancer initiated a campaign under the name “MIND THE GAP” to help increase awareness and minimize the discrepancies associated with lung cancer. Its main purpose is to improve the health literacy of the Greek population regarding lung cancer risk factors and screening methods, in addition to fighting the stigma associated with this disease, with the ultimate goal being to serve the underprivileged. This campaign has already gained international status, with the Greek Society of Lung Cancer presenting its purposes at the 5th European Cancer Forum in Brussels. It should be ensured that beating cancer will continue to be a priority for Europe.

## 6. Conclusions

It is nowadays well established that there are socioeconomic inequalities regarding lung cancer incidence, screening, effective treatment, overall survival, and prognosis. Treatments regarding lung cancer have improved for the general population but have worsened for underrepresented populations. The battle against lung cancer is relentless. It is time that pivotal actions towards narrowing the disparities in treatment and patient care are initiated in an effort to ensure that prioritizing the defeat of cancer is not only a priority for Europe but for the entire world.
